# Supplementary material for: Specific Gene Duplication and Loss of Cytochrome P450 in Families 1-3 in Carnivora (Mammalia, Laurasiatheria)
Source: Animals (Basel). 2022 Oct 18;12(20):2821. doi: 10.3390/ani12202821 (PMC9597770; doi:10.3390/ani12202821)
Supplement: Supplementary file 1 [file animals-12-02821-s001.zip › Supplemental Data.pdf]

Supplemental Data

Supplemental Figure S1. Synteny of Pinniped CYP3As.

Sequence files: CYP2C_protein.fas, CYP3A_protein.fas.

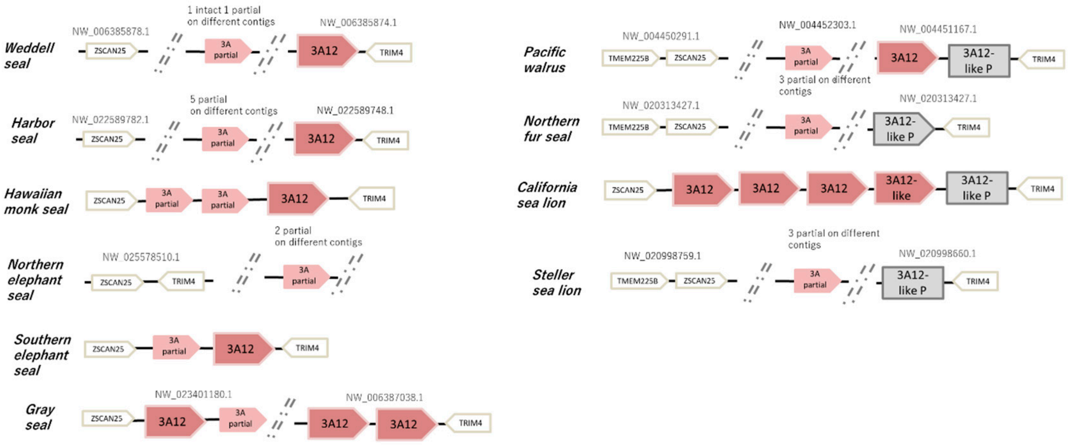

Supplemental table S1. Sequence information of CYP2Cs

Transcript	Gene name	Species name
XM027062670.1	(LOC106965834)	Acinonyx jubatus
XM015061917.2	(LOC106965832)	Acinonyx jubatus
XM019809407.2	(LOC100468534)	Ailuropoda melanoleuca
XM002931139.4	(LOC100471407)	Ailuropoda melanoleuca
XM002927756.4	(LOC100470213)	Ailuropoda melanoleuca
XM025881012.1	(LOC112830847)	Callorhinus ursinus
XM025880701.1	(LOC112830695)	Callorhinus ursinus
XM025880212.1	(LOC112830415)	Callorhinus ursinus
XM025880084.1	(LOC112830321)	Callorhinus ursinus
XM038441028	(LOC100688697)	Canis lupus familialis
NM001197044	(CYP2C18)	Canis lupus familiaris
NM001003334	(CYP2C41)	Canis lupus familiaris
NM001003339	CYP2E1	Canis lupus familiaris
XM022499564.1	(LOC111144947)	Enhydra lutris
XM022499543.1	(LOC111144935)	Enhydra lutris
XM028120002.1	(LOC114221836)	Eumetopias jubatus
XM028098977.1	(LOC114205474)	Eumetopias jubatus
XM028098724.1	(LOC114205328)	Eumetopias jubatus
XM019813570.3	(LOC101089804)	Felis catus
XM019813568	(CYP2C41)	Felis catus
NM001048012	LOC554344	Felis catus
NM001048010	CYP2E1	Felis catus
XM036111709	LOC118547938	H. grypus
XM036111713.1	(LOC118547943)	Halichoerus grypus
NM000771.4	CYP2C9	Homo sapiens
NM000770.3	CYP2C8	Homo sapiens
NM000769.4	CYP2C19	Homo sapiens
NM000772.3	CYP2C18	Homo sapiens
NM000773	CYP2E1	Homo sapiens

XM039230221	(LOC120230594)	Hyaena hyaena
XM039230218.1	(LOC120230589)	Hyaena hyaena
XM039230216.1	(LOC120230588)	Hyaena hyaena
XM006735587	LOC102742186	L. weddelli
XM045438286.1	(LOC123576853)	Leopardus geoffroyi
XM045438283.1	(LOC123576852)	Leopardus geoffroyi
XM006735585.2	(LOC102741683)	Leptonychotes weddellii
XM032835939.1	(LOC116854234)	Lontra canadensis
XM032835932.1	(LOC116854212)	Lontra canadensis
XM032835932.1	(LOC116854212)	Lontra canadensis
XM047703224.1	(LOC125085077)	Lutra lutra
XM030335026.1	(LOC115527426)	Lynx canadensis
XM030334984.1	(LOC115527383)	Lynx canadensis
XM045878464	LOC123846700	M. angustirostris
XM035025489	LOC118024011	M. leonina
XM046027750.1	(LOC123955564)	Meles meles
XM046027685.1	(LOC123955523)	Meles meles
XM046027649	LOC123955513	Meles meles
XM045878463.1	(LOC123846699)	Mirounga angustirostris
XM035025476.1	(LOC118024006)	Mirounga leonina
XM032313320.1	(LOC116573275)	Mustela erminea
XM032313316.1	(LOC116573274)	Mustela erminea
XM004749633.2	(LOC101688450)	Mustela putorius
XM032313316.1	(LOC101687589)	Mustela putorius
XM021699489	LOC110589090	N. schauinslandi
XM021699824.1	(LOC110589295)	Neomonachus schauinslandi
XM044236635.1	(LOC122898982)	Neovison vison
XM044236636.1	(LOC122898983)	Neovison vison
XM004416880.2	(LOC101383285)	Odobenus rosmarus
XM004412554.1	(LOC101365655)	Odobenus rosmarus
XM004394967.1	(LOC101365057)	Odobenus rosmarus
XM032399170	LOC116628835	P. vitulina
XM042908866.1	(LOC122202477)	Panthera leo
XM042908683.1	(LOC122202356)	Panthera leo
XM019445145.1	(LOC109264682)	Panthera pardus
XM019445142.1	(LOC109264681)	Panthera pardus
XM015536661.2	(LOC102958169)	Panthera tigris
XM015536660	(LOC102957879)	Panthera tigris
XM032432414.1	(LOC116649523)	Phoca vitulina
XM032399170.1	(LOC116628835)	Phoca vitulina
XM043597027.1	(LOC122493053)	Prionailurus bengalensis
XM043597025.1	(LOC122493051)	Prionailurus bengalensis
XM025917875.1	(LOC112854424)	Puma concolor
XM025917868.1	(LOC112854419)	Puma concolor
XM040485921.1	(LOC121037025)	Puma yagouaroundi
XM040485917.1	(LOC121037024)	Puma yagouaroundi
NM031839	Cyp2c23	Rattus norvegicus
XM039104416	Cyp2c11	Rattus norvegicus
NM001271354	Cyp2c24	Rattus norvegicus
XM039096704	LOC100912265	Rattus norvegicus
XM039101412	LOC120093077	Rattus norvegicus
NM017158	Cyp2c7	Rattus norvegicus

XM039098183	LOC100911718	Rattus norvegicus
XM039098189	LOC120098584	Rattus norvegicus
XM039108994	Cyp2c6v1	Rattus norvegicus
NM031572	Cyp2c12	Rattus norvegicus
NM138514	Cyp2c13	Rattus norvegicus
XM029916398.1	(LOC115273367)	Suricata suricatta
XM029916374.1	(LOC115273354)	Suricata suricatta
XM044382289	LOC113252105	Ursus actos
XM048218837	LOC113243099	Ursus actos
XM048218836	LOC125282749	Ursus actos
XM048218835	LOC113251748	Ursus actos
XM045809991.1	(LOC123800347)	Ursus americanus
XM045809363.1	(LOC123800006)	Ursus americanus
XM045795979.1	(LOC123791300)	Ursus americanus
XM045785073.1	(LOC123783994)	Ursus americanus
XM045785072.1	(LOC123783992)	Ursus americanus
XM045785070.1	(LOC123783991)	Ursus americanus
XM040624282.1	(LOC103659697)	Ursus maritimus
XM008710147.2	(LOC103680656)	Ursus maritimus
XM008699299.2	(LOC103670756)	Ursus maritimus
XM040624282	LOC103659697	Ursus maritimus
XM041746082	(LOC121485560)	Vulpes lagopus
XM041745448.1	(LOC121485285)	Vulpes lagopus
XM041744155	(LOC121484666)	Vulpes lagopus
XM025995655.1	(LOC112917654)	Vulpes vulpes
XM025995642	(LOC112917651)	Vulpes vulpes
XM027596314.1	(LOC113923499)	Zalophus californianus
XM027595941.1	(LOC113923367)	Zalophus californianus
XM027588917.1	(LOC113919561)	Zalophus californianus
Plar01080		Paguma larvata

Supplemental table S2. Sequence information of CYP3As

Transcript	Gene	Species name
XM15079337.2	(LOC106981197)	Acinonyx jubatus
XM15079336.2	(LOC106981196)	Acinonyx jubatus
XM25861581	(LOC112815559)	Callorhinus ursinus
XM38668135.1	(CYP3A4)	Canis lupus
XM38668133.1	(LOC119876349)	Canis lupus
XM38668129.1	(LOC119875773)	Canis lupus
XM38538847.1	(LOC489851)	Canis lupus
NM1003340.1	(CYP3A12)	Canis lupus
NM1003338.1	(CYP3A26)	Canis lupus
NM 212673	cyp3c1	Daniorerio
XM 21474709	cyp3c2	Daniorerio
XM 28096741	LOC114203387	E.jubatus
XM 28097104	LOC114203807	E.jubatus
XM 28096948	LOC114203610	E.jubatus
XM22500615	(LOC111145608)	Enhydra lutris
XM22500610.1	(LOC111145602)	Enhydra lutris
XM22500609.1	(LOC111145601)	Enhydra lutris
NM1246271.1	(CYP3A132)	Felis catus

AB558978.1	CYP131	Felis catus
XM36078737.1	(LOC118527106)	Halichoerus grypus
XM36078736.1	(LOC118527105)	Halichoerus grypus
XM36064786.1	(LOC118518203)	Halichoerus grypus
NM 1202855	CYP3A4	Homo sapiens
XM39226068	(LOC120227978)	Hyaena hyaena
XM39225508	(LOC120227384)	Hyaena hyaena
XM39223729	(LOC120225539)	Hyaena hyaena
XM45460912.1	(LOC123589186)	Leopardus geoffroyi
XM31021941	(LOC102725583)	Leptonychotes weddellii
XM32872634.1	(LOC116876456)	Lontra canadensis
XM32872624.1	(LOC116876447)	Lontra canadensis
XM32872312	(LOC116876260)	Lontra canadensis
XM30300402.1	(LOC115503873)	Lynx canadensis
XM45996924.1	(LOC123936278)	Meles meles
XM45996922.1	(LOC123936277)	Meles meles
XM45992661	(LOC123933470)	Meles meles
XM45992644.1	(LOC123933458)	Meles meles
XM45992643.1	(LOC123933457)	Meles meles
XM45992642.1	(LOC123933456)	Meles meles
XM45992631.1	(LOC123933455)	Meles meles
XM34988445	(LOC117999383)	Mirounga leonina
XM32328302.1	(LOC116581214)	Mustela erminea
XM32328297.1	(LOC116581213)	Mustela erminea
XM32328295.1	(LOC116581212)	Mustela erminea
XM45075078	(LOC101681834)	Mustela putorius
XM45075076.1	(LOC101676428)	Mustela putorius
XM45075072.1	(LOC101676727)	Mustela putorius
XM45075071.1	(LOC101682130)	Mustela putorius
XM44915717.1	(LOC110571974)	Neomonachus schauinslandi
XM44232711.1	(LOC122895360)	Neovison vison
XM44232710.1	(LOC122895359)	Neovison vison
XM44232704	(LOC122895351)	Neovison vison
XM44232699.1	(LOC122895350)	Neovison vison
	Plar09515mRNA	Paguma larvata
	Plar08536mRNA	Paguma larvata
	Plar08537mRNA	Paguma larvata
XM42922685.1	(LOC122210161)	Panthera leo
XM42922633.1	(LOC122210147)	Panthera leo
XM19422667.1	(LOC109251250)	Panthera pardus
XM19422666.1	(LOC109251249)	Panthera pardus
XM7081063.3	(LOC102957670)	Panthera tigris
XM7081062.3	(CYP3A131)	Panthera tigris
XM32430882.1	(LOC116648714)	Phoca vitulina
XM43561244.1	(LOC122471976)	Prionailurus bengalensis
XM43561242.1	(LOC122471975)	Prionailurus bengalensis
XM40456280.1	(LOC121017809)	Puma yagouaroundi
XM40456279.1	(LOC121017808)	Puma yagouaroundi
XM4414732	LOC101368110	rosmarus
XM29946900.1	(LOC115298293)	Suricata suricatta
XM29945742	(LOC115297553)	Suricata suricatta
XM29945739	(LOC115297551)	Suricata suricatta

XM 48224737	LOC113249444	U.arctos
XM 48224738	LOC113249757	U.arctos
XM 48224740	LOC113267902	U.arctos
XM45773906.1	(LOC123777328)	Ursus americanus
XM45773818.1	(LOC123777277)	Ursus americanus
XM45773797.1	(LOC123777260)	Ursus americanus
XM26516220.2	(LOC113267901)	Ursus arctos
XM8698388.2	(LOC103669999)	Ursus maritimus
XM8698387.2	(LOC103669998)	Ursus maritimus
XM8698381.2	(LOC103669994)	Ursus maritimus
XM41750336.1	(LOC121488071)	Vulpes lagopus
XM41747127	(LOC121486274)	Vulpes lagopus
XM26019628	(LOC112935745)	Vulpes vulpes
XM26019624	(LOC112935742)	Vulpes vulpes
XM25989350	(LOC112912717)	Vulpes vulpes
XM25986193	(LOC112909983)	Vulpes vulpes
XM35722054.1	(LOC113932071)	Zalophus californianus
XM35722053.1	(LOC118355996)	Zalophus californianus
XM35722052.1	(LOC113932025)	Zalophus californianus
XM35722051.1	(LOC118355995)	Zalophus californianus

Table S3. Food habitats of Carnivoran.

Species	Food habitats	References
Acinonyx jubatus	**Carnivore**	[69,70]
Ailuropoda melanoleuca	**Herbivore**	[36,71]
Callorhinus ursinus	**Fishivore**	[72–75]
Canis lupus familiaris	**Omnivore**	[76–78]
Crocuta crocuta	**Insectivore, Omnivore**	[79,80]
Enhydra lutris kenyoni	**Fishivore**	[75,81]
Eumetopias jubatus	**Fishivore**	[75,82]
Felis catus	**Carnivora**	[83,34]
Halichoerus grypus	**Fishivore**	[84]
Hyaena hyaena	**Carnivore**	[85,86]
Leptonychotes weddellii	**Fishivore**	[75,87]
Lontra canadensis	**Fishivore**	[88,89]
Lutra lutra	**Fishivore**	[90]
Lynx canadensis	**Carnivora**	[91,92]
Meles meles	**Insectivore, Omnivore**	[33,93]
Mirounga angustirostris	**Fishivore**	[94]
Mirounga leonina	**Fishivore**	[95,96]
Mustela erminea	**Carnivora**	[97,98]
Mustela putorius furo	**Carnivora**	[99,100]
Neogale vison	**Carnivora**	[101–103]
Neomonachus schauinslandi	**Fishivore**	[75,104]
Odobenus rosmarus divergens	**Fishivore**	[105,106]
Panthera leo	**Carnivora**	[117,108]
Panthera pardus	**Carnivora**	[109,110]
Panthera tigris	**Carnivora**	[111,112]
Phoca vitulina	**Fishivore**	[75,113]
Prionailurus bengalensis	**Carnivora**	[115,116]

Prionailurus viverrinus	**Carnivora**	[114]
Puma concolor	**Carnivora**	[116,117]
Puma yagouaroundi	**Carnivora**	[118,119]
Ursus americanus	**Omnivore**	[31,120–122]
Ursus arctos	**Omnivore**	[30,123–125]
Ursus maritimus	**Carnivore**	[124,125]
Vulpes lagopus	**Omnivore**	[126,127]
Vulpes vulpes	**Omnivore**	[128,129]
Zalophus californianus	**Fishivore**	[130,131]

Supplemental table S4. Assemble and annotation information.

Assembly accession number, species scientific names, and annotation information used in this study are shown.

Assembly Accession	Organism Name	Annotation Name
GCF_003709585.1	Acinonyx jubatus	NCBI Annotation Release 101
GCF_002007445.2	Ailuropoda melanoleuca	NCBI eukaryotic genome annotation pipeline
GCF_003265705.1	Callorhinus ursinus	NCBI Annotation Release 100
GCF_014441545.1	Canis lupus familiaris	NCBI Annotation Release 106
GCA_008692635.1	Crocuta crocuta	Annotation submitted by BGI
GCF_002288905.1	Enhydra lutris kenyoni	NCBI Annotation Release 100
GCF_004028035.1	Eumetopias jubatus	NCBI Annotation Release 100
GCF_018350175.1	Felis catus	NCBI Annotation Release 105
GCF_012393455.1	Halichoerus grypus	NCBI Annotation Release 100
GCF_003009895.1	Hyaena hyaena	NCBI Annotation Release 100
GCF_000349705.1	Leptonychotes weddellii	NCBI Annotation Release 101
GCF_010015895.1	Lontra canadensis	NCBI Annotation Release 100
GCF_902655055.1	Lutra lutra	NCBI Annotation Release 100
GCF_007474595.2	Lynx canadensis	NCBI Annotation Release 102
GCF_022079265.1	Lynx rufus	NCBI Annotation Release 100
GCF_922984935.1	Meles meles	NCBI Annotation Release 100
GCF_021288785.1	Mirounga angustirostris	NCBI Annotation Release 100
GCF_011800145.1	Mirounga leonina	NCBI Annotation Release 100
GCF_009829155.1	Mustela erminea	NCBI Annotation Release 100
GCF_011764305.1	Mustela putorius furo	NCBI Annotation Release 102
GCF_020171115.1	Neogale vison	NCBI Annotation Release 100
GCF_002201575.2	Neomonachus schauinslandi	NCBI Annotation Release 101
GCF_000321225.1	Odobenus rosmarus divergens	NCBI Annotation Release 101
GCF_018350215.1	Panthera leo	NCBI Annotation Release 100
GCF_001857705.1	Panthera pardus	NCBI Annotation Release 100
GCF_018350195.1	Panthera tigris	NCBI eukaryotic genome annotation pipeline
GCF_004348235.1	Phoca vitulina	NCBI Annotation Release 100
GCF_016509475.1	Prionailurus bengalensis	NCBI Annotation Release 100
GCF_022837055.1	Prionailurus viverrinus	NCBI Annotation Release 100
GCF_003327715.1	Puma concolor	NCBI Annotation Release 100
GCF_014898765.1	Puma yagouaroundi	NCBI Annotation Release 100
GCF_006229205.1	Suricata suricatta	NCBI Annotation Release 100
GCF_020975775.1	Ursus americanus	NCBI Annotation Release 100
GCF_023065955.1	Ursus arctos	NCBI eukaryotic genome annotation pipeline

GCF_017311325.1	Ursus maritimus	NCBI Annotation Release 101
GCF_018345385.1	Vulpes lagopus	NCBI Annotation Release 100
GCF_003160815.1	Vulpes vulpes	NCBI Annotation Release 100
GCF_009762305.2	Zalophus californianus	NCBI Annotation Release 101
